# An Analysis of Capsaicin, Dihydrocapsaicin, Vitamin C and Flavones in Different Tissues during the Development of Ornamental Pepper

**DOI:** 10.3390/plants13152038

**Published:** 2024-07-24

**Authors:** June Wang, Xudong Duan, Yu An, Jinyao He, Jiaxin Li, Jingqi Xian, Daofen Zhou

**Affiliations:** Shanxi Key Laboratory of Germplasm Resources Innovation and Utilization of Vegetable and Flower, College of Horticulture, Shanxi Agricultural University, Jinzhong 030801, China; 18735427450@163.com (X.D.); anyu625625@163.com (Y.A.); hejinyao98@163.com (J.H.); 13283666922@163.com (J.L.); 15534431864@163.com (J.X.); 18386635976@163.com (D.Z.)

**Keywords:** ornamental pepper, capsaicin, high-performance liquid chromatography (HPLC), principal component analysis (PCA), qPCR

## Abstract

As a fruit and vegetable crop, the ornamental pepper is not just highly ornamental but also rich in nutritional value. The quality of ornamental pepper fruits is given in their contents of capsaicin, vitamin C (VC), flavonoids and total phenols. The study concentrated on the accumulation of capsaicin and dihydrocapsaicin in different tissues of 18 peppers during fruit growth and development. The results showed that the pericarp and placenta contained significantly higher levels of capsaicin than dihydrocapsaicin. Additionally, the placenta contained significantly higher levels of both capsaicin and dihydrocapsaicin compared to the pericarp. The content of capsaicin was in the range of 0–6.7915 mg·g^−1^, the range of dihydrocapsaicin content was 0–5.329 mg·g^−1^. Interestingly, we found that the pericarp is rich in VC (5.4506 mg·g^−1^) and the placenta is high in flavonoids (4.8203 mg·g^−1^) and total phenols (119.63 mg·g^−1^). The capsaicin is the most important component using the correlation analysis and principal component analysis. The qPCR results substantiated that the expression of genes in the placenta was significantly higher than that in the pericarp and that the expression of genes in green ripening stage was higher than that in red ripening stage. This study could be utilized to select the best ripening stages and tissues to harvest peppers according to the use of the pepper and to the needs of producers. It not only provides a reference for quality improvement and processing for consumers and market but also provides a theoretical basis for high-quality pepper breeding.

## 1. Introduction

The ornamental pepper (*Capsicum annuum* L.) is a kind of ornamental leaf and fruit vegetable, which belongs to the plant family Solanaceae. The ornamental pepper has high ornamental and application value because of its unique fruit shape and gorgeous color. It can not only be used with ornamental flowers but also for landscaping and the beautification of the environment [1]. Like traditional peppers, ornamental peppers have high nutritional values. The fruit is rich in capsaicin, VC and other nutrients [2]. These substances are important indexes to evaluate the nutritional quality of plants. The contents of nutritional components, vitamins and capsaicin from different cultivars are different [3]. Capsaicin is a unique and important component in peppers that is beneficial to human health. It has been applied in the medical industry [4,5]. In addition to the pungency of pepper fruits, it is also characterized by high VC content [6]. Regarding other nutritional qualities, it contributes to human health, such as reducing obesity and diabetes [7].

The spicy taste of capsaicin is determined by the content of capsaicin, which is unique to the fruit of capsaicin, and it is respected and loved by people for its medical and health care functions, such as anti-cancer, analgesic and itching properties, in the medical field [8]. Capsaicin and dihydrocapsaicin are the major alkaloids, which account for 90% of the total capsaicinoids in peppers [9,10]. The biosynthesis of capsaicin compounds is present in the placenta of capsicum [11]. Capsaicin compounds accumulate in vacuoles in the placental epidermis until cell metabolism occurs, and then, extracellular secretion occurs. In recent studies, it was found that capsaicinoid biosynthesis also occurs in the pericarp of extremely pungent cultivars [12]. The quantification of capsaicin and dihydrocapsaicin performed by HPLC-PDA on selected peppers found the highest amount in the Naga pepper and the lowest in the paprika pepper [13]. The content of capsaicin varies in the different developmental stages of peppers. In the case of the pericarp, the maximum capsaicinoid concentration was reached at 30 days post-anthesis (dpa) (with values of 1565.98 and 2158.82 µmol·kg^−1^ for the young and adult plant, respectively), while in the placenta, it was registered at 41 dpa with greater values (5898.12 and 2349.67 µmol·kg^−1^) [14]. Materska reported a comparison of the placenta and pericarp of capsicum fruits, with the placenta being the most abundant in flavonoids and the pericarp having a greater diversity of glycosylated compounds. Up until now, however, little is known about the tissue-specific spatiotemporal location of other compounds and secondary metabolites in capsicum fruits [15]. Although capsaicin has been well characterized in previous studies, there is a lack of comprehensive analyses covering two different dimensions of tissue sites and the development stage of capsaicin.

The capsaicinoid biosynthesis pathway is composed of the phenylpropanoid and branched-chain fatty acid pathways [16]. At present, the enzymes involved in capsaicin synthesis that have been reported include capsaicin synthase (*CS*), cinnamic acid hydrolase (*C4H*), caffeoyl-CoA O-methyltransferase (*CoMT*), β-ketoacyl-ACP synthase (*KAS*) and other enzymes [17]. The study found a significant positive correlation between the activity of *CS* and *PAL* and the capsaicin content. Therefore, *CS* and *PAL* are generally considered to be the key enzymes for capsaicin synthesis [18]. In the process of capsaicin synthesis, *AT*, *CoMT*, *KAS*, *C4H*, *AMT* and other structural genes are associated with the synthesis of capsaicin [19]. Studies have shown that AT3 (*Pun1*) controls the synthesis of capsaicin and the spiciness of peppers [20]. Understanding the accumulation pattern of capsaicin content in fruits of different genotypes can provide a theoretical basis for the biosynthesis and accumulation of capsaicin fruits. The characterized pepper materials can provide a valuable reference for the genetic improvement of pepper spiciness.

VC, flavonoids and total phenols were closely connected with the appearance, flavor and nutritional quality of pepper. Peppers have been shown to be a rich source of VC, which helps reduce the risk of many types of cancer and heart disease [21]. VC maintains important biological properties and antioxidant capacity [22]. A number of studies have shown that total phenols and total flavonoids are directly related to the antioxidant activity of plants and play a role in promoting the antioxidant activity of plants [23]. Sweet peppers are rich in polyphenols, such as p-coumaric, ferulic, p-hydroxybenzoic, caffeic acid and sinapic acid and quercetin-3-glucoside [24]. Although a number of studies have reported the growth changes of capsicum and the corresponding differences in the content of major components, there are limited studies on the optimal site and period of accumulation of this bioactive compound during the fruit development of different cultivars. Observing the changes of different parts in the whole development period of pepper fruits can not only enhance our understanding of the fruit growth patterns but also improve the quality of pepper.

The purpose of this study was to comprehensively and systematically study the contents of capsaicin, dihydrocapsaicin, VC, flavonoids and total phenols in the placenta and pericarp of capsicum at different developmental stages and determine the key growth time points and tissues. The key factors for evaluating the nutritional quality of capsicum were determined by principal component analysis. The expression of genes related to the capsaicin synthesis pathway in different cultivars and tissues at different growth stages were analyzed. The difference in capsaicin and its metabolite content in different cultivars may be helpful for pepper breeding. This study takes into account the state of the tissue of the fruit during ripening, at which stage the concentration of the bioactive compounds of interest to us reaches its maximum. The aim is to provide the basis for breeding pepper cultivars with good nutritional quality and lay the foundation for planting, developing and applying edible peppers.

## 2. Results

### 2.1. Analysis of Capsaicin and Dihydrocapsaicin Contents in Different Ornamental Peppers

The content of capsaicin in the pericarp ranged from 0.0074 mg·g^−1^FW to 1.1982 mg·g^−1^FW. The highest was the green ripening fruit of ‘M3’, but the content dropped rapidly in the advanced stage. By comparing the accumulation of capsaicin in the pericarp at different periods, we explored that ‘Q3’, ‘S1’, ‘W5’, ‘W8’, ‘Wu’ and ‘Y1’ have higher accumulation in the green ripening stage. At the red ripening stage, the content of capsaicin in the pericarp for ‘Q2’ was the highest, and the lowest accumulation in capsaicin was for ‘W2’, which was almost undetectable. We found a higher accumulation in the placenta than the pericarp, which ranged from 0.0123 mg·g^−1^FW to 6.7915 mg·g^−1^FW. At the green ripening stage, the highest content of capsaicin in the placenta was ‘B7’ (6.6310 mg·g^−1^), followed by ‘W8’ (6.5867 mg·g^−1^), and the lowest was ‘W2’ (0.0123 mg·g^−1^). At the red ripening stage, the content of capsaicin for ‘C1’ was the highest (6.7915 mg·g^−1^), and the content of capsaicin for ‘W4’ was the lowest (0.0641 mg·g^−1^). The content of capsaicin in the red ripening placenta of ‘C1’, ‘C2’, ‘M3’, ‘Q2’, ‘Q3’, ‘W5’ and ‘Y1’ was higher than that in the green ripening placenta.

**Figure 1:**
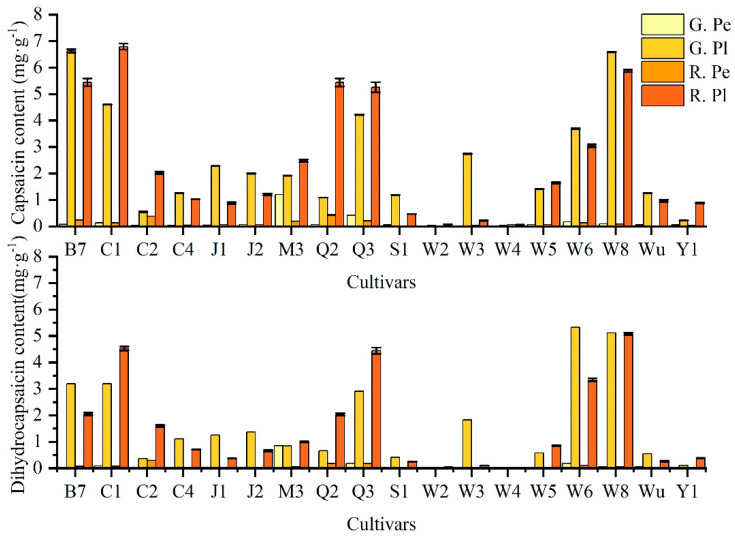
The contents of capsaicin and dihydrocapsaicin in eighteen pepper cultivars at the green and red stage and two different tissues (pericarp and placenta). Note: R. Pl refers to the placenta at the red ripening stage. R. Pe refers to the pericarp at the red ripening stage. G. Pl refers to the placenta at the green ripening stage. G. Pe refers to the pericarp at the green ripening stage. The following is the same. Data are the means of three biological replications ± SE.

The highest content of dihydrocapsaicin in the pericarp was 0.8507 mg·g^−1^ at the green ripening stage of ‘M3’, while the content of dihydrocapsaicin in the pericarp was only 0.0592 mg·g^−1^ at the red ripening stage. The contents of dihydrocapsaicin in the pericarp of ‘W2’ and ‘W3’ were quite insignificant during the growth and development of fruits. ‘C2’ (0.3006 mg·g^−1^) had the highest content of dihydrocapsaicin in the pericarp at the red ripening stage. ‘Wu’ and ‘Y1’ accumulated a small amount of dihydrocapsaicin in the green ripening stage, but it was almost decomposed at the red ripening stage. The ‘W6’ in the green ripening stage showed the most dihydrocapsaicin (5.3293 mg·g^−1^), followed by ‘W8’, ‘B7’ and ‘C1’, with 5.1193 mg·g^−1^, 3.1933 mg·g^−1^ and 3.1908 mg·g^−1^, respectively (Figure 1).

### 2.2. Analysis of Main Nutritional Quality of Different Ornamental Peppers

**Figure 2:**
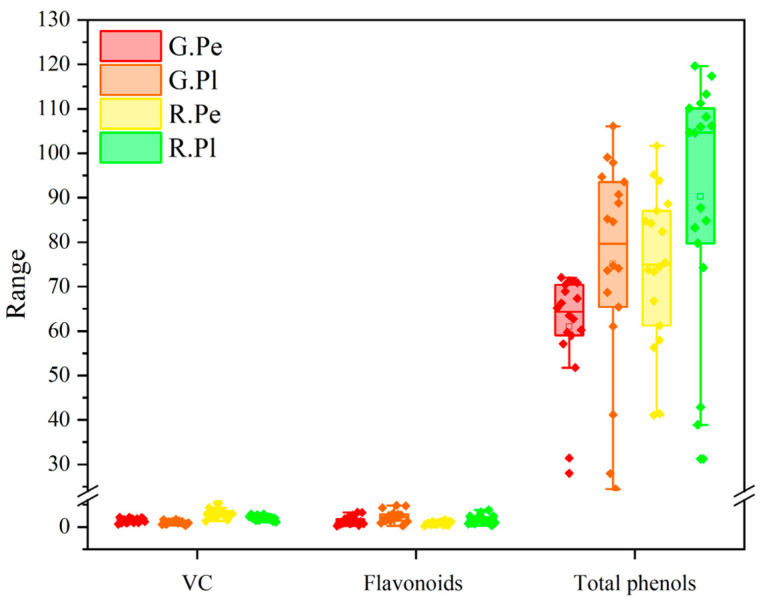
Contents of VC, flavonoids and total phenols in eighteen pepper cultivars at green and red stage and two different tissues (pericarp and placenta).

The nutritional quality of the pericarp and placenta cultivars was analyzed in the growth and development of 18 pepper cultivars (Figure 2). It can be observed that the total phenol content is higher compared to the VC and flavonoid content. There is a significant difference in the VC content between the green ripening stage and red ripening stage, with most cultivars showing a higher VC content in the red ripening stage. The pericarp exhibits higher VC content than the placenta, with the ‘C4’ red ripening stage having the highest concentration (5.4506 mg·g^−1^). Conversely, the ‘W4’ green ripening stage shows the lowest concentration in the placenta (0.3308 mg·g^−1^). Flavonoids are important components of plants, and the contents of flavonoids in different cultivars of peppers showed different trends during the growth and development stage. The difference was obvious in different tissues. The flavonoid content in the placenta was higher than that in the pericarp, and the flavonoid content in the placenta at the ‘C2’ green maturation stage was the highest (4.8203 mg·g^−1^). It is worth noting that the contents of total phenols and flavonoids showed the same trend; specifically, the total phenol content of the placenta was higher than that of the pericarp.

### 2.3. Correlation Analysis and Principal Component Analysis of Components in Different Developmental Stages of Ornamental Pepper

**Figure 3:**
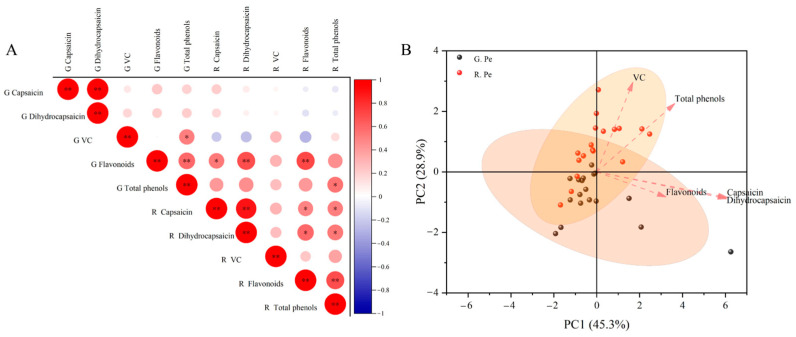
The correlation (**A**) and principal component analysis (**B**) of the components in the pericarp of ornamental peppers at different developmental stages. Note: * and ** indicate a significant correlation (*p* < 0.05) and very significant correlation (*p* < 0.01), respectively. The following is the same.

In order to explore the relationship of components in different periods of the pepper pericarp, the correlation analysis of components in the green ripening stage and red ripening stage was carried out (Figure 3A). The results showed that the content of capsaicin in the green ripening stage was significantly correlated with the content of dihydrocapsaicin in the green ripening stage (*p* < 0.01), and the content of flavonoids in the green ripening stage was significantly correlated with the content of total phenols in the green ripening stage, dihydrocapsaicin in the red ripening stage and flavonoids in the red ripening stage (*p* < 0.01). There was significant correlation between the capsaicin and dihydrocapsaicin content in the red ripening stage (*p* < 0.01), and there was significant correlation between the flavonoid content and total phenol content in the red ripening stage (*p* < 0.01). A PCA analysis was performed according to the content of components in the pepper pericarp at different periods. According to the content of the components in the capsicum pericarp at different periods, the PCA analysis showed that the contribution rates of PC1 and PC2 were 45.3% and 28.9%, respectively (Figure 3B). The five components had a positive correlation to PC1, capsaicin and dihydrocapsaicin contributed the most to PC1, VC and total phenols had a positive correlation to PC2, but capsaicin, dihydrocapsaicin and flavonoids had a negative correlation to PC2.

**Figure 4:**
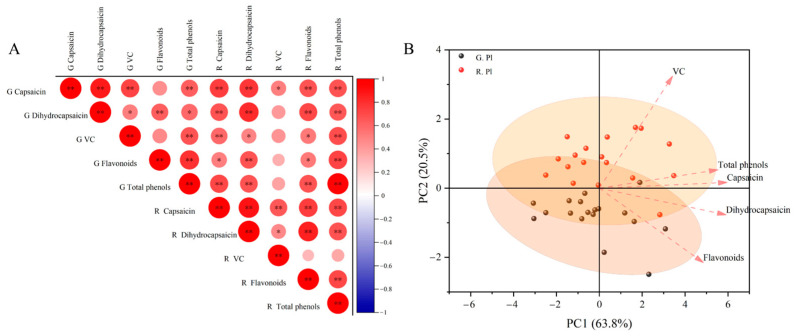
Correlation (**A**) and principal component analysis (**B**) of components in placenta of ornamental pepper at different developmental stages. Note: * and ** indicate a significant correlation (*p* < 0.05) and very significant correlation (*p* < 0.01), respectively. The following is the same.

Through the correlation analysis of the content of the components in the placenta of the green ripening stage and red ripening stage, it was found that the content of capsaicin in the green ripening stage was significantly correlated with the content of other components except for the content of VC in the green ripening stage and red ripening stage (Figure 4A). Dihydrocapsaicin in the green ripening stage was significantly correlated with the content of flavonoids in the green ripening stage, capsaicin in the red ripening stage, dihydrocapsaicin in the red ripening stage, flavonoids in the red ripening stage and total phenols in the red ripening stage (*p* < 0.01). VC content in the green ripening stage was significantly correlated with the total phenol content in the green ripening stage, capsaicin in the red ripening stage and the total phenol content in the red ripening stage, and flavonoids in the green ripening stage were significantly correlated with the total phenol content in the green ripening stage, dihydrocapsaicin in the red ripening stage and the total phenol content in the red ripening stage. There was a significant correlation between total phenols in the green ripening stage and the nutrient content in the red ripening stage, except the VC content (*p* < 0.05). There was a significant correlation between capsaicin and other nutrients in the red ripening stage (*p* < 0.01). There was a significant correlation between dihydrocapsaicin in the red ripening stage and flavonoids in the red ripening stage and the total phenol content in the red ripening stage and a significant correlation between flavonoids in the red ripening stage and the total phenol content in the red ripening stage (*p* < 0.05). The PCA analysis showed that the contribution rate of PC1 and PC2 was 63.8% and 20.5%, respectively (Figure 4B). Five components were positively correlated with PC1, among which capsaicin and dihydrocapsaicin contributed the most to PC1, VC and capsaicin; total phenols were positively correlated with PC2, and dihydrocapsaicin and flavonoids were negatively correlated with PC2.

### 2.4. Correlation Analysis and Principal Component Analysis of Components in Different Tissue Parts of Ornamental Pepper

**Figure 5:**
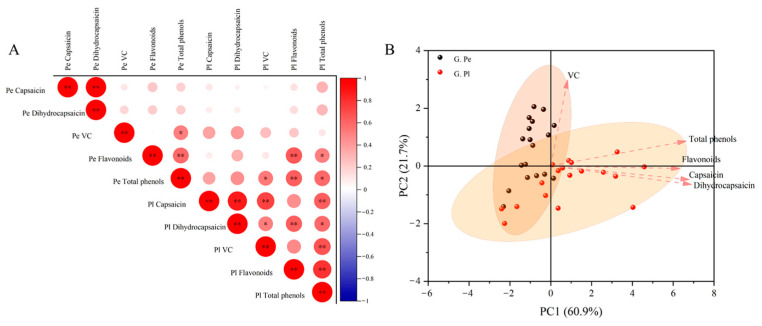
Correlation analysis (**A**) and principal component analysis (**B**) of components in different tissue parts of ornamental pepper during green ripening stage. Note: * and ** indicate a significant correlation (*p* < 0.05) and very significant correlation (*p* < 0.01), respectively. The following is the same.

The correlation of nutritional quality in different parts of ornamental peppers at the green ripening stage was analyzed (Figure 5A). The content of capsaicin in the pericarp was positively correlated with that of dihydrocapsaicin in the pericarp (*p* < 0.01). The flavonoid content in the pericarp was positively correlated with the flavonoid content of total phenols in the pericarp (*p* < 0.01), and the capsaicin content in the placenta was positively correlated with the content of dihydrocapsaicin, VC in the placenta and total phenols in the placenta (*p* < 0.01). The content of flavonoids in the placenta was positively correlated with the content of dihydrocapsaicin in the placenta and total phenols in the pericarp (*p* < 0.01). The total phenols in the placenta was positively correlated with VC and the flavonoid content in the placenta (*p* < 0.01). There were significant correlations among other parameters as well (*p* < 0.05). The results of our PCA show that the contribution of the first principal component (PC1) and the second principal component (PC2) is 60.9% and 21.7%, respectively (Figure 5B), indicating that they cover the comprehensive information of most parameters. In addition to VC, the other four indicators have a greater contribution to PC1. Interestingly, the content of components in the pericarp of ornamental capsicum during the green ripening stage is mainly VC, and the content of components in the pericarp during the green ripening stage is closely related to capsaicin and dihydrocapsaicin.

**Figure 6:**
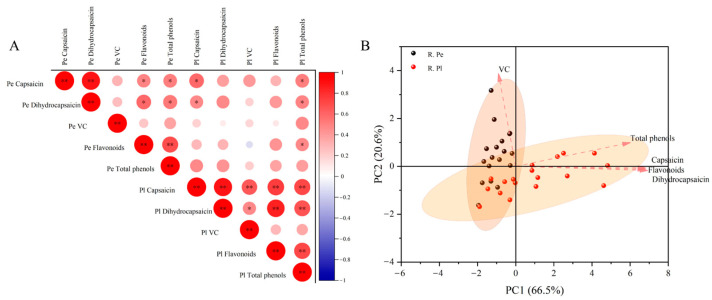
Correlation analysis (**A**) and principal component analysis (**B**) of components in different tissue parts of ornamental pepper during red ripening stage. Note: * and ** indicate a significant correlation (*p* < 0.05) and very significant correlation (*p* < 0.01), respectively. The following is the same.

The correlation of nutritional quality in different parts of the ornamental capsicum red ripening stage was further analyzed (Figure 6A). The content of capsaicin in the pericarp was positively correlated with that of dihydrocapsaicin (*p* < 0.01). The flavonoid content in the pericarp was positively correlated with the total phenolic flavonoid content in the pericarp (*p* < 0.01), and the capsaicin content in the placenta was positively correlated with other nutrients’ content in the placenta (*p* < 0.01). The content of dihydrocapsaicin in the placenta was positively correlated with the content of flavonoids and total phenols in the placenta (*p* < 0.01). There was a significant positive correlation between the total phenol and flavonoid content in the placenta (*p* < 0.01). The results of our principal component analysis show that the contribution of the first principal component (PC1) and the second principal component (PC2) is 66.5% and 20.6%, respectively (Figure 6B), indicating that they cover the comprehensive information of most parameters. In addition to VC, the other four indicators contribute more to PC1. Different from the green ripening stage, VC and PC1 showed a large negative correlation.

### 2.5. Analysis of Expression Patterns of Genes Related to Capsaicin Synthesis

**Figure 7:**
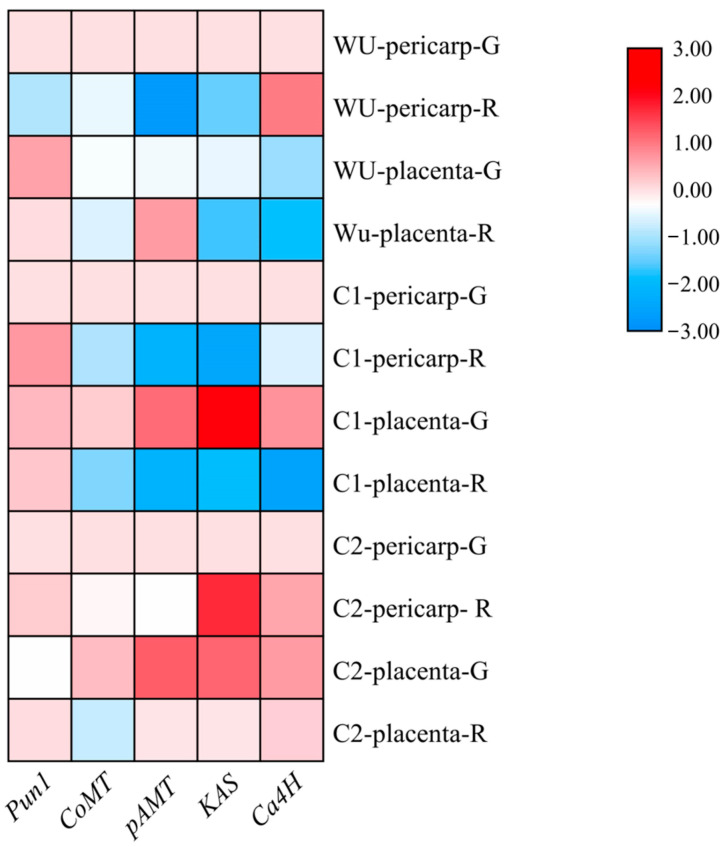
Heat map showing capsaicinoid biosynthetic pathway (*Pun1*, *CoMT*, *KAS*, *C4H*, *AMT*) genes in three pepper cultivars (‘Wu’, ‘C1’ and ‘C2’) at green and red stage and two different tissues (pericarp and placenta).

The expression patterns of genes related to the capsaicin synthesis pathway (*Pun1*, *CoMT*, *pAMT*, *KAS*, *Ca4H*) were analyzed (Figure 7). The results showed that the expression of each gene was significantly higher in ‘C2’ than in the other cultivars. The expression of *Pun1* in the pericarp and placenta of ‘Wu’ was lower at the red ripening stage than at the green ripening stage, while in the pericarp of ‘C1’, the expression of *Pun1* was up-regulated after green ripening, i.e., the expression of *Pun1* was higher at the red ripening stage than at the green ripening stage, and the expression of *Pun1* was higher at the green ripening stage than at the red ripening stage in the placenta of ‘C1’. The expression of *CoMT* in the three peppers followed the same trend, and the expression of *CoMT* was higher at the green ripening stage than at red ripening. The expression trend of *pAMT* in the pericarp and placenta of ‘Wu’, ‘C1’ and ‘C2’ was the same as that of *CoMT*, with higher expression at the green ripening stage than at the red ripening stage. The expression of *KAS* in the pericarp and placenta of ‘Wu’ and ‘C1’ had the same trend, i.e., the expression was higher at the green ripening stage than at the red ripening stage. In the placenta of ‘C2’, the expression of *KAS* was higher at the green ripening stage than at the red ripening stage, while in its pericarp, the opposite was true, with the expression of *KAS* being significantly higher at the red ripening stage than at the green ripening stage. In the pericarp of ‘C2’, *Ca4H* showed an up-regulation of expression at the red ripening stage compared to the green ripening stage, whereas in the placentas of the three cultivars and in the pericarp of ‘Wu’ and ‘C1’, the opposite trend was observed, with higher expression at the green ripening stage, followed by a down-regulation of *Ca4H* expression.

**Figure 8:**
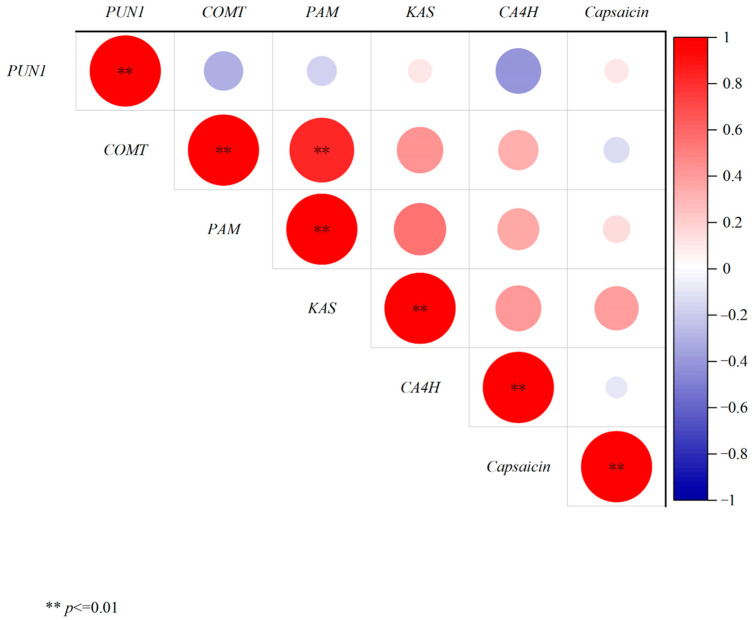
Correlation analysis results for the capsaicin contents and the expression levels of capsaicin synthesis-related genes.

It was conducted on the correlation between the expression levels of capsaicin and capsaicin synthesis-related genes in different developmental stages and tissues of different cultivars (Figure 8). The results showed that the correlation between the capsaicin content and *Pun1*, *PAM*, *KAS*, *COMT* and *CA4H* was not significant, but the capsaicin content was positively correlated with the expression levels of *Pun1*, *PAM* and *KAS*, with correlation coefficients of 0.11513, 0.15242 and 0.38804, respectively. There is a negative correlation with the expression levels of *COMT* and *CA4H*. The correlation coefficients are −0.13563 and −0.09593, respectively.

## 3. Discussion

The biosynthesis of capsaicin is tissue specific. CAP biosynthesis is limited to the placental septum of fruits, but it has been reported that its biosynthesis occurs even in the pericarp of some extremely pungent cultivars, resulting in a substantial increase in the total content [25]. In the present study, we also found that the content of capsaicin and dihydrocapsaicin in the placenta was higher than that in the pericarp. The biosynthesis of capsaicin is spatially–temporally specific. For different cultivars, the stage of capsaicin production and rapid accumulation was different. Generally, the synthesis of capsaicin starts around 15 d after flowering and increases gradually with the maturity of the fruit. It reaches the peak at the turning stage or red ripening stage [19,26,27]. The capsaicin concentration in the placenta and pericarp of Jeromin peppers reached its maximum at the 40th and 60th dpa [28]. We simultaneously analyzed different tissues (pericarp and placenta) and two stages of plant maturity (30 and 50 days after flowering) and found consistent changes in the capsaicin content in the placenta of ‘C1’, ‘C2’, ‘Q2’ and ‘Q3’. The capsaicin content in the red maturing stage (50 d after flowering) was higher than that in the green maturing stage (30 d after flowering). However, the capsaicin content in the placentation of some cultivars in this study was higher at 30 d after flowering (green ripening stage) than at 50 d after flowering (red ripening stage), such as ‘B7’, ‘J1’, ‘J2’, ‘W6’, ‘W8’ and ‘Wu’. It has also been demonstrated that capsaicin compounds reach their maximum on 30 d after flowering in seedlings, while in adult plants, capsaicin compounds reach their maximum concentration in the placenta and pericarp on 40 d and 60 d, respectively [28,29]. Previous studies have focused on the capsaicin content at different tissues and at different stages of ripening in a single pepper cultivar, whereas others have focused on capsaicin content at the stage of fruit ripening or different tissues in a few (3–4) peppers. It has been shown that the concentration of bioactive compounds in each pepper variety follows a different accumulation pattern throughout the ripening process [30]. This study analyzed 18 pepper cultivars and speculated that the above results were caused by the variety and type of pepper.

Capsaicin is a unique alkaloid in peppers, but the quality of peppers is also determined by vitamin C, flavonoids and total phenols. The ripening stage had a significant difference in the bioactive compounds studied in the present work (VC, flavonoids and total phenols); this behavior was demonstrated by other works. Ripe peppers showed the highest concentration of vitamin E (9.69 ± 0.02 mg/100 g of dry mass) and vitamin C (119.44 ± 4.72 mg/100 g of dry mass) [31]. The total phenolics, flavonoids and total anthocyanin contents of the red pepper lines were in the range of 7.06–17.15 mg gallic acid equivalent (GAE)/g dw, 1.10–5.46 mg catechin equivalent (CE)/g dw and 7.9–516.6 mg/kg dw extract, respectively [32]. The total phenol content (17.38–131.5 mg GAE/g dry weight) and total flavonoid content (14.07–56.15 mg quercetin/g dry weight) of 45 genotypes were determined [33]. We found that the current research on the accumulation of these bioactive compounds in different tissue parts during the development of different cultivars of peppers was limited, so we analyzed them. Interestingly, the VC content in the pericarp was higher than that in the placenta, among which the VC content in the ‘C4’ red maturation stage was the highest (5.4506 mg·g^−1^), and the content of flavonoids and total phenols in the placenta was higher than that in the pericarp.

To investigate the correlation between nutritional quality emerged as a mandatory task [34]. We further investigated the correlation of the quality characteristics of various component quality features in two tissue parts of 18 cultivars of capsicum during two developmental stages. In addition, the principal component analysis method was used to analyze it comprehensively. The PCA was performed to provide an overall analysis of the 11 morphological and quality features of the five cultivars of goji berries during the maturation process; the total sugar and polysaccharide can be identified as the main evaluation factors for the five goji berry cultivars during the four development stages [35]. A deeper understanding of the taste differences among various parts of the wampee fruit was achieved through a PCA. The cumulative variance contribution from PC1 (67.5%) and PC2 (29.1%) reached an impressive 96.6%. The seed, pulp and peel formed distinct clusters, reflecting their different taste profiles [36]. In this study, a PCA was used to analyze the quality characteristics of two tissues during the ripening process of 18 cultivars of capsicum. Capsaicin and dihydrocapsaicin were the main evaluation factors.

According to the results of the principal component analysis, the gene expression profile related to the capsaicin synthesis pathway of conventional cultivars was analyzed. The key genes in the synthesis pathway of capsaicin were strongly expressed at 16–20 days after flowering, which made the capsaicin substances increase at an earlier stage and then decrease at the harvest stage [37]. On the other hand, the rapid increase and peak time of capsaicin were also related to cultivars and affected by environmental factors [38]. The expression level of genes related to capsaicin synthesis in the placenta was higher than that in pericarp maturation, reached the highest expression level around 40 DPA and showed a downward trend [39]. This was consistent with the results of this experiment, as the expression levels of *Pun1*, *CoMT*, *pAMT*, *KAS* and *Ca4H* in the placenta of each pepper variety were significantly higher than those in the pericarp. In addition, it was found that the expression of all genes in the green ripening stage was higher than that in the red ripening stage. *pAMT* was expressed in the placental compartment of ripe green fruits [40]. In this study, *pAMT* also showed the same trend of change. This may be because these genes are early biosynthetic genes, so the expression level of *pAMT* in the green ripen stage is higher than that in the red ripen stage. The expression level in the pericarp of some pepper cultivars in the green ripening stage was lower than that in the red ripening stage, which may be due to the different reactions of different materials to external environmental factors (temperature, rain, etc.) so that the synthesis and decomposition of capsaicin were carried out at the same time, resulting in different trends in the expression of related genes. The specific reasons need to be further studied. *Pun1* was highly expressed in the placenta of spicy peppers and reached its maximum value at 20 days after flowering, while the expression level of *Pun1* was extremely low in sweet peppers, which was consistent with the results of this study, and the expression level of *Pun1* was higher at the green ripening stage [41,42]. By silencing *Pun1* in peppers, resulted in reduced capsaicinoid contents in fruits [43,44]. This suggests that *Pun1* plays an important role in the synthesis of capsaicin. The Kondo study found that seven genes (*ACS*, *PAL*, *C4H*, *4CL*, *C3H*, *HCT* and *COMT*) were not significantly associated with capsaicin concentration, which is consistent with our findings [45]. The study hypothesizes that this is because capsaicin synthesis is regulated by multiple genes, not just by these five genes. In addition, the content of capsaicin should be related to the total expression of genes related to capsaicin synthesis during the growth and development of capsaicin rather than the high gene expression in a certain period, the high capsaicin content. The expression products of genes are enzymes, and environmental conditions have a great influence on the activity of enzymes, which may also be the cause of this result [46]. Therefore, the next step should be to conduct a comprehensive analysis along with enzyme activity measurements to enhance the scientific validity of the test.

## 4. Materials and Methods

### 4.1. Plant Material

**Figure 9:**
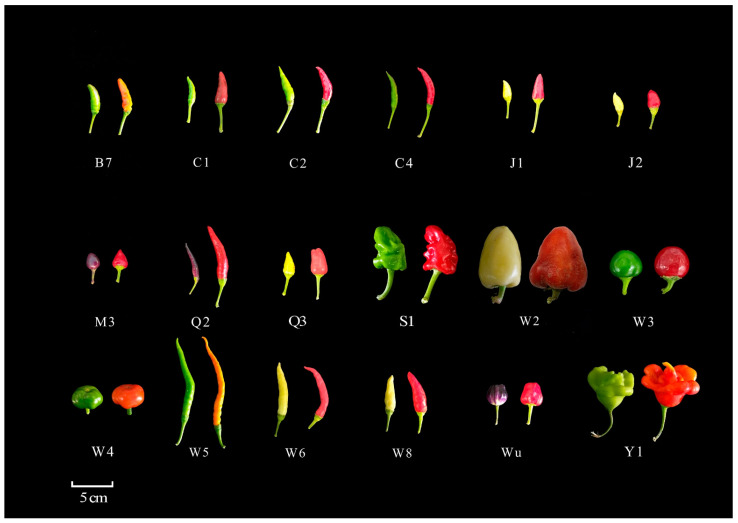
The testing material. Note: B7. *Capsicum annuum* L. var. *dactylus* M.; C1. *Capsicum annuum* L. var. *cerasiforme* Irish.; C2. *Capsicum annuum* L. var. *fascic ulatum* sturt.; C4. *Capsicum annuum* L. var. *fascic ulatum* sturt.; J1. *Capsicum annuum* L. var. *dactylus* M.; J2. *Capsicum annuum* L. var. *cerasiforme* Irish.; M3. *Capsicum annuum* L. var. *cerasiforme* Irish.; Q2. *Capsicum annuum* L. var. *dactylus* M.; Q3. *Capsicum annuum* L. var. *Annuum.*; S1. *Capsicum annuum* L. var. *grossum* Sent.; W2. *Capsicum annuum* L. var. *breviconcidum* Haz.; W3. *Capsicum annuum* L. var. *cerasiforme* Irish.; W4. *Capsicum annuum L. var. breviconcidum Haz.*; W5. *Capsicum annuum* L. var. *dactylus* M.; W6. *Capsicum annuum* L. var. *dactylus* M.; W8. * Capsicum annuum* L. var. *cerasiforme* Irish.; Wu. *Capsicum annuum* L. var. *cerasiforme* Irish.; Y1. *Capsicum annuum* L. var. *breviconcidum* Haz.

Eighteen pepper cultivars were cultivated in the horticultural experimental station of Shanxi Agricultural University (Figure 9). Pepper fruits were sampled at 2 different stages during fruit ripening (30 and 50 days after flowering). The pericarp and placenta were separated and stored in the refrigerator at −80 °C for quality determination. Pepper cultivars ‘C1’, ‘C2’ and ‘Wu’ were tested to analyze the expression patterns of capsaicin synthesis-related genes.

### 4.2. Chemicals and Reagents

The capsaicinoid reference standards, capsaicin (CAS: 404-86-4, HPLC ≥ 98%) and dihydrocapsaicin (CAS:19408-84-5, HPLC ≥ 98%) were purchased from Solarbio (Beijing Solarbio Science&Technology Co., Ltd., Beijing, China). The methanol used for the extraction and chromatographic separation, HPLC grade, were supplied by (Tianjin Kemiou Chemical Reagent Co., Ltd., Tianjin, China). Folin–Ciocalteu’s phenol reagent was of a biological reagent (F6011G, Biotopped, Beijing, China). Other reagents were of analytical grade. Eihylenediamine tetraacetic acid disodium salt and ammonium molybdate were provided by Fengchuan (Tianjin Fengchuan Chemical Reagent Co., Ltd., Tianjin, China). Metaphosphoric acid, aluminum nitrate and L-ascorbic acid were provided by Kemiou (Tianjin Kemiou Chemical Reagent Co., Ltd., Tianjin, China). Oxalic acid dihydrate, sodium nitrite, sodium carbonate anhydrous and sodiumhydroxide were provided by Kaitong (Tianjin Kaitong Chemical Reagent Co., Ltd., Tianjin, China). Sulfuric acid was provided by Chron Chemicals (ChengDu Chron Chemicals Co., Ltd., Chengdu, China)

### 4.3. Determination of Capsaicin and Dihydrocapsaicin

The sample was ground into a powder with liquid nitrogen, 0.25 g was taken into 5 mL methanol for 30 min with ultrasound assisted extraction (SB25-12DTD, NingBo Scientz Biotechnology Co., Ltd., Ningbo, China) and centrifuged at 4000 rpm for 5 min (HC-2518R, Anhui Ustc Zonkia Scientific Instruments Co., Ltd., Hefei, China) and the supernatant was transferred to a 10 mL tube. The supernatant collected twice was combined; the methanol was constant volume to 10 mL and then subjected to 0.22 mL organic filter membrane for chromatographic analysis (Thermo Fisher U3000 HPLC, Waltham, MA, USA).

Chromatographic conditions: C18 (5 µm, 4.6 mm × 250 mm, Dikma Technologies Inc., Beijing, China), Mobile phase: methanol/Ultra-pure water: 70/30, Flow rate: 1.0 mL·min^−1^, Detection wavelength: 280 nm, Sample injection volume: 10 L, Column temperature: 35 °C.

### 4.4. Determination of Nutrient Content

The content of VC was determined by ammonium molybdate colorimetry [47]. A sample (0.2 g) was ground in 2.5 mL oxalate-EDTA extract. The pepper pulp was centrifuged at 8000 rpm for 10 min, and the supernatant was collected. An amount of 1 mL supernatant was mixed with 3 mL oxalate-EDTA extract, 0.5 mL metaphosphoric acid-acetic acid solution, 1 mL 5% H_2_SO_4_ and 2 mL 5% ammonium molybdate solution, respectively, and placed in 30 °C water bath for 15 min (HH-2, Changzhou Langyue Instrument Manufacturing Co., Ltd., Changzhou, China). The flavonoid content was determined by sodium nitrite–aluminum nitrate method, and the total phenol content was determined by Folin–Ciocalteu [48,49]. An amount of 0.5 g sample was added with 4 mL 80% ethanol; the extraction was carried out at 40 °C for 30 min by ultrasonic-assisted extraction. After centrifugation at 4 °C and 9000 rpm for 10 min, the supernatant was collected. Then, we repeated the above operation at constant volume of 10 mL. An amount of 1 mL of supernatant was absorbed, 0.25 mL 5% NaNO_2_ was added, it was shaken well and we let it stand for 6 min. Then, we added 0.25 mL 10% Al (NO_3_)_3_ and let it stand for 6min. Then, 1.5 mL 4% NaOH was added, and the flavonoid content was measured after standing for 15 min. In addition, we took 1.0 mL sample solution and diluted it 5 times, then diluted 0.5 mL of Folin–Ciocalteu 4 times, and 2 mL of 10% Na_2_CO_3_ solution were successively added. The total phenol content was determined after standing for 1 h. According to the absorption values of VC, flavonoids and total phenols at wavelengths 760, 510 and 760 nm, the contents were determined by spectrophotometer (UV-2600 Shimadzu, Kyoto, Japan).

### 4.5. RNA Extraction, cDNA Synthesis and Real-Time RT-qPCR

RNA was extracted using a modified protocol of Guo [1] and cDNA was obtained according to the instructions of PrimeScript™ RT reagent kit with gDNA Eraser (Takara, Beijing, China). The cDNA synthesized by reverse transcription was used as a template for PCR amplification with reference to the TB Green^®^ Premix Ex Taq™ II kit (Takara, China) operating instructions, and the expression level was calculated using 2^−ΔΔCT^.

The nucleotide sequences of *Pun1*, *CoMT*, *pAMT*, *KAS* and *Ca4H* in capsicum plants were searched and compared on NCBI (https://www.ncbi.nlm.nih.gov/, accessed on 5 July 2020). The primers (Table 1) were designed using SnapGene 4.2.4 and synthesized by Sangon Biotech (Shanghai, China).

### 4.6. Statistical Analysis

The experiment for each sample was repeated three times, and difference analysis, principal component analysis and correlation analysis between indexes were carried out by SPSS 23.0. The plots were drawn with Origin 2021 and Excel 2010.

## 5. Conclusions

In the present study, the nutritional characteristics of different tissues and different developmental stages of capsicum were comprehensively analyzed. The key growth time point was determined to be 50 days after flowering. We found that the capsaicin content in the pericarp and placenta was significantly higher than that of dihydrocapsaicin, and the content of capsaicin and dihydrocapsaicin content in the placenta was also significantly higher than that in the pericarp. The content of capsaicin was in the range of 0–6.7915 mg·g^−1^, the range of dihydrocapsaicin content was 0–5.329 mg·g^−1^. In addition, we confirmed that the pericarp is rich in VC, up to 5.4506 mg·g^−1^. The placenta is rich in total phenols (119.63 mg·g^−1^) and flavonoids (4.8203 mg·g^−1^) and has certain biological activities. Therefore, these two tissues can be processed to make full use of their nutritional value. We also found a significant correlation between the nutritional quality of pepper at different developmental stages and different tissues. Capsaicin and dihydrocapsaicin were used as the two main evaluation factors in the principal component analysis. The expression levels of genes related to the synthesis pathway of capsaicin were analyzed. The expressions of *Pun1*, *CoMT*, *pAMT*, *KAS* and *Ca4H* in the placenta were significantly higher than those in the pericarp. In addition, the expression of all genes showed a trend of green maturity higher than red maturity. This study can not only provide a theoretical basis for the harvest stage and organization of peppers but also provide convenience for the further cultivation and breeding of peppers.

## Figures and Tables

**Table 1:** Primer sequence.

Genes	Accession No.	Primer Sequence	Length (bp)
*Pun1*	GU300812.1	F:GTGATGGTTGCTCTCTGCTT R:GTGGCGTAATGAGAGAACCA	144
*CoMT*	AF081214.1	F:GAGCCTAACTCAAACAGAGGA R:GGTCAAGTTCTACGGTTGCTT	101
*pAMT*	AF085149.1	F:TCTCTCTGGGCTTCCTCCAA R:TCTTCGGTTTCACCTGGCAA	111
*KAS*	AF085148.1	F:ACGGACGCTTTTATTTCGGC R:CGGAGCCTTCACCAATGACA	139
*Ca4H*	AF088847.1	F:CCGCTCACTGGAAGAAACC R:TCCTCCTACCAACACCGAA	117

## Data Availability

The original contributions presented in the study are included in the article/Supplementary Materials, further inquiries can be directed to the corresponding authors.

## Supplementary Materials

The following supporting information can be downloaded at https://www.mdpi.com/article/10.3390/plants13152038/s1, Figure S1: Calibration curve, (A) regression equation for Capsaicin, (B) regression equation for Dihydrocapsaicin; Figure S2: Calibration curve, (a) regression equation for flavonoid contents, (b) regression equation for total phenol contents, (c) regression equation for VC; Figure S3: HPLC test for capsaicin and dihydrochilin standards; Figure S4: Determination of capsaicin and dihydrocapsaicin in ornamental pepper samples by HPLC.
