# Supplementary material for: An Analysis of Capsaicin, Dihydrocapsaicin, Vitamin C and Flavones in Different Tissues during the Development of Ornamental Pepper
Source: Plants (Basel). 2024 Jul 24;13(15):2038. doi: 10.3390/plants13152038 (PMC11313734; doi:10.3390/plants13152038)
Supplement: Supplementary file 1 [file plants-13-02038-s001.zip › plants-3078925-supplementary.pdf]

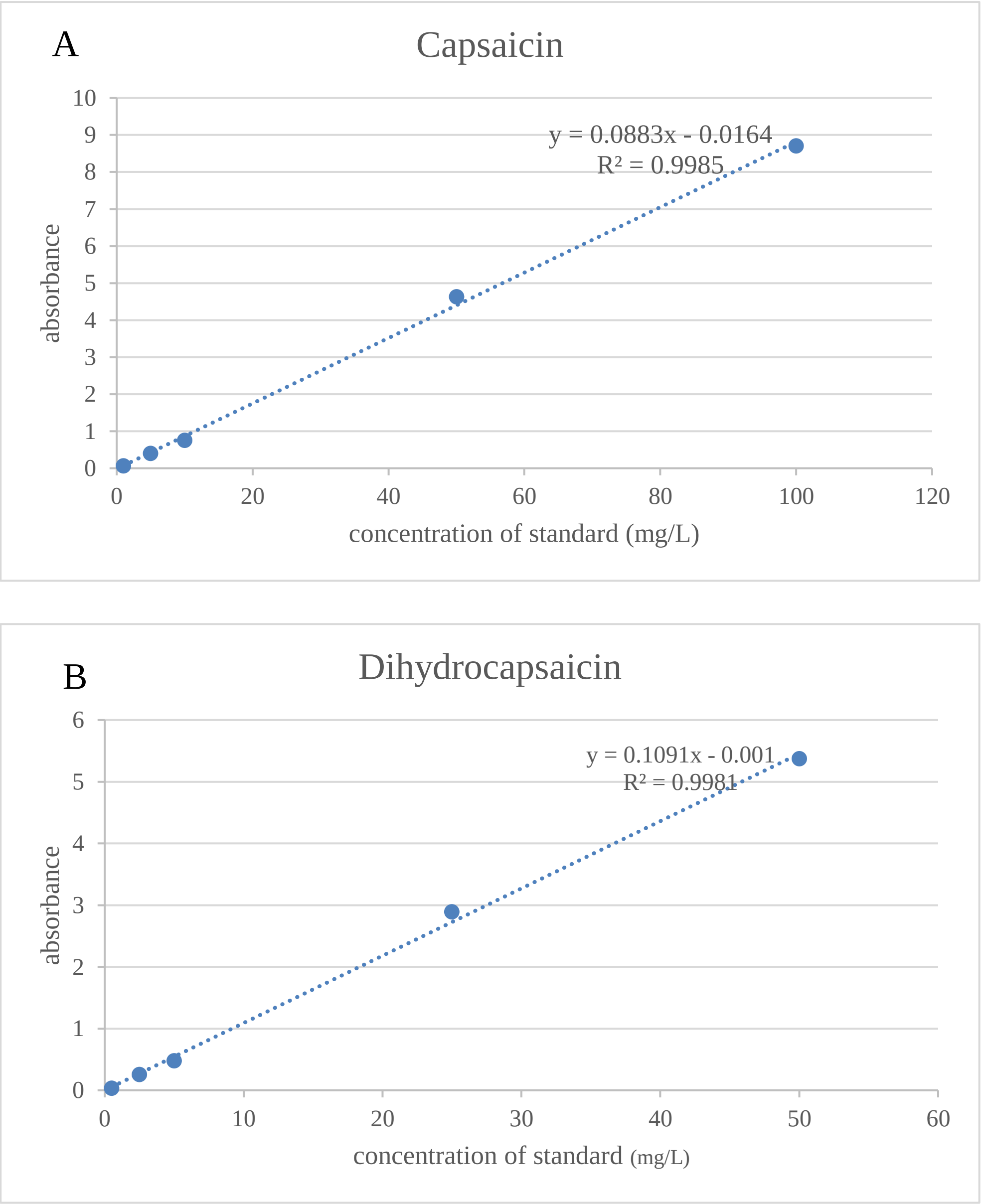

Figure S1. Calibration curve, (**A**) regression equation for Capsaicin, (**B**) regression equation for Dihydrocapsaicin

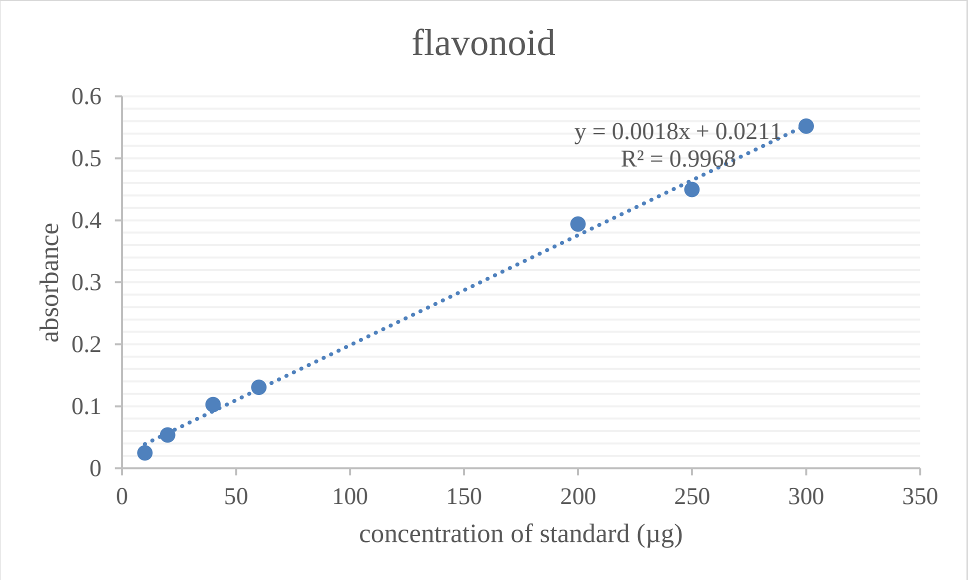
flavonoid
y = 0.0018x + 0.0211
R² = 0.9968
0.6
0.5
0.4
0.3
0.2
0.1
0
absorbance
0
50
100
150
200
250
300
350
concentration of standard (µg)

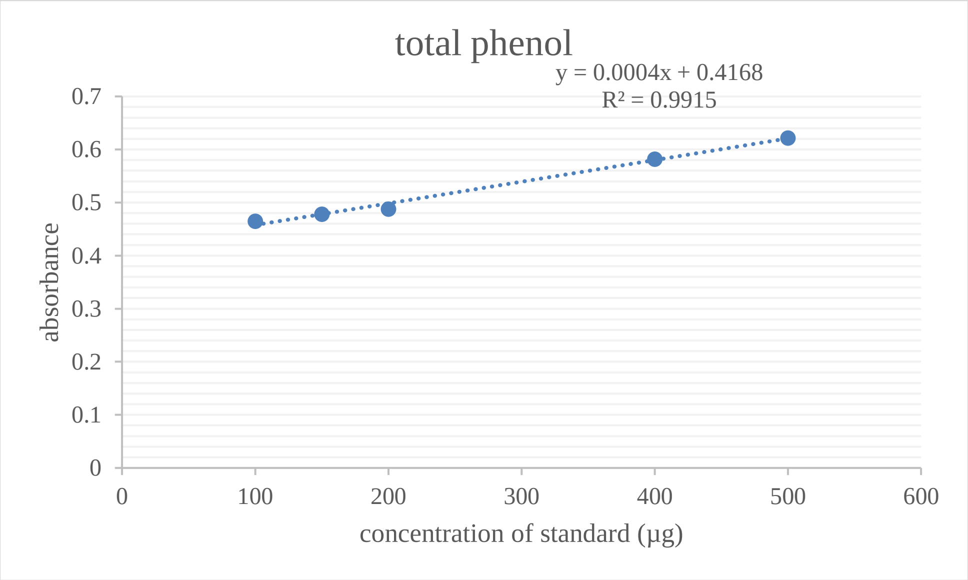
total phenol
y = 0.0004x + 0.4168
R² = 0.9915
0.7
0.6
0.5
0.4
0.3
0.2
0.1
0
absorbance
0
100
200
300
400
500
600
concentration of standard (µg)

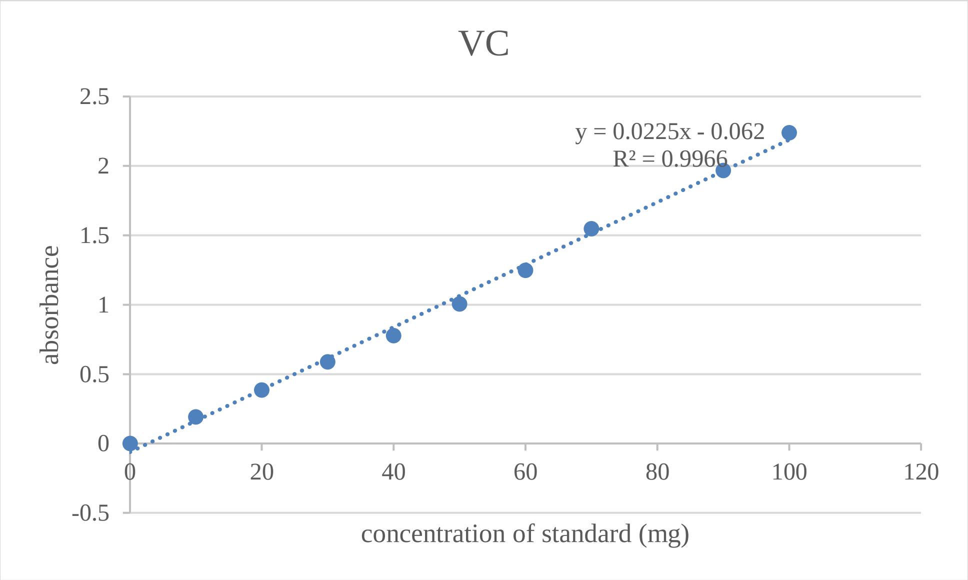
VC
y = 0.0225x - 0.062
R² = 0.9966
2.5
2
1.5
1
0.5
0
-0.5
absorbance
0
20
40
60
80
100
120
concentration of standard (mg)

Figure S2. Calibration curve, (a) regression equation for flavonoid contents, (b) regression equation for total phenol contents, (c) regression equation for VC

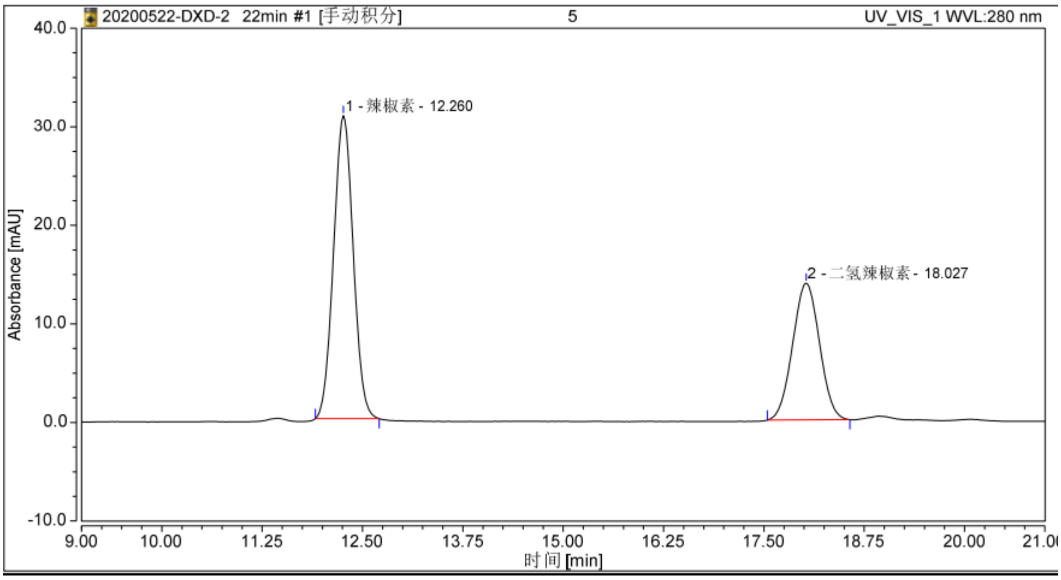

Figure S3 HPLC test for capsaicin and dihydrochilin standards

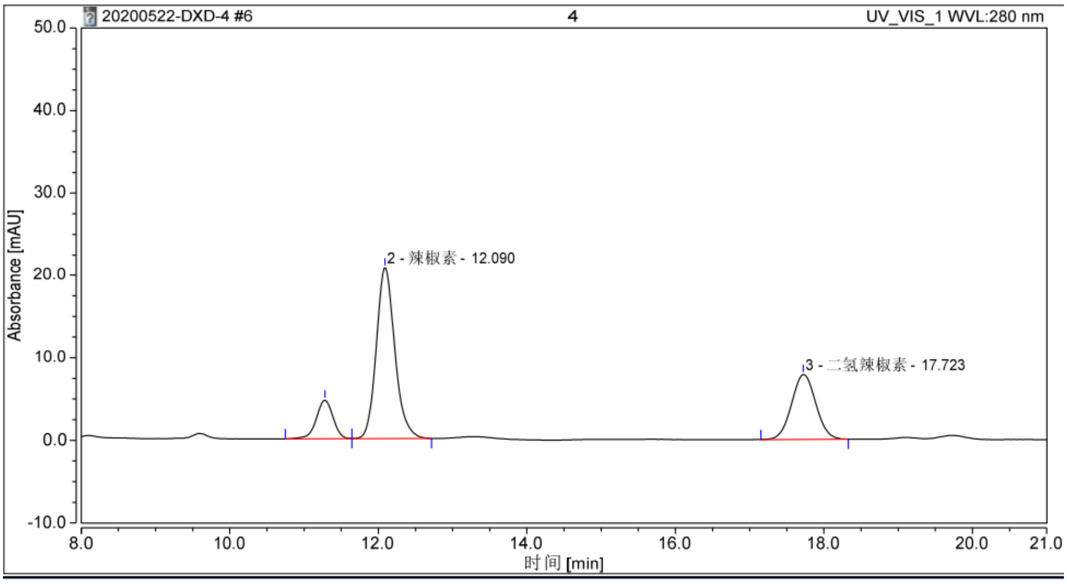

Figure S4 Determination of capsaicin and dihydrocapsaicin in ornamental pepper samples by HPLC
